# Symptom severity of patients with advanced cancer in palliative care unit: longitudinal assessments of symptoms improvement

**DOI:** 10.1186/s12904-016-0105-8

**Published:** 2016-03-11

**Authors:** Shu-Yu Tai, Chung-Yin Lee, Chien-Yi Wu, Hui-Ya Hsieh, Joh-Jong Huang, Chia-Tsuan Huang, Chen-Yu Chien

**Affiliations:** Graduate Institute of Medicine, College of Medicine, Kaohsiung Medical University, Kaohsiung, Taiwan; Department of Family Medicine, School of Medicine, College of Medicine, Kaohsiung Medical University, Kaohsiung, Taiwan; Department of Family Medicine, Kaohsiung Medical University Hospital, Kaohsiung, Taiwan; Department of Family Medicine, Kaohsiung Municipal Ta-Tung Hospital, Kaohsiung Medical University, Kaohsiung, Taiwan; Department of Nursing, Kaohsiung Medical University Hospital, Kaohsiung Medical University, Kaohsiung, Taiwan; Department of Otorhinolaryngology, School of Medicine, College of Medicine, Kaohsiung Medical University, Kaohsiung, Taiwan; Departments of Otorhinolaryngology, Kaohsiung Medical University Hospital, Kaohsiung, Taiwan; Department of Otolaryngology, Kaohsiung Municipal Hsiao-Kang Hospital, Kaohsiung Medical University, Kaohsiung, Taiwan

**Keywords:** Advanced cancer, Palliative care, Symptom severity, Generalised estimating equation

## Abstract

**Background:**

This study assessed the symptom severity of patients with advanced cancer in a palliative care unit and explored the factors associated with symptom improvement.

**Methods:**

This study was conducted in a palliative care unit in Taiwan between October 2004 and December 2009. Symptom intensity was measured by the “Symptom Reporting Form”, and graded on a scale of 0 to 4 (0 = *none*, and 4 = *extreme*). These measures were assessed on the 1^st^, 3^rd^, 5^th^, and 7^th^ Day in the palliative care unit. The study data comprised routine clinical records and patients’ demographic data. Generalized estimating equation (GEE) was used to assess the symptom improvement, and investigate the factors associated with the symptom reporting form scores.

**Results:**

Among the 824 recruited patients with advanced cancer, pain (78.4 %), anorexia (64.4 %) and constipation (63.5 %)were the most common and severe symptom. After controlling for other factors in the multivariate GEE model, the day of palliative care administration was a significant factor associated with all of the scales, except Days 7 on the dyspnoea and oedema scales and Day 5 on the anxiety scale. In addition, patients aged ≥ 65 years exhibited significantly lower scores on the pain, sleep disturbance, depression, and anxiety scales than did those aged < 65 years. Moreover, female patients exhibited higher scores on the vomiting, anorexia, oedema, depression, and anxiety scales than did male patients. Furthermore, patients with gastrointestinal tract cancer exhibited higher scores on the constipation, vomiting, anorexia, oedema, depression, and anxiety scales and lower scores on the dyspnoea scale than did those with lung cancer. Patients with breast cancer exhibited higher scores on the oedema scale and lower scores on the anxiety scale. Patients with genitourinary cancer exhibited higher scores on the vomiting and oedema scales and lower scores on the dyspnoea scale. Patients with head, neck, and oral cancer exhibited lower scores on the oedema scale alone.

**Conclusion:**

The symptom severity declined during the first week in the palliative care unit. In addition, differences in sex and primary cancer sites may contribute to varying degrees of symptom improvement.

**Electronic supplementary material:**

The online version of this article (doi:10.1186/s12904-016-0105-8) contains supplementary material, which is available to authorized users.

## Background

Cancer is a leading cause of death worldwide and accounted for 8.2 million deaths in 2012 [1]. Since 1982, cancer has been the primary cause of death in Taiwan and accounts for over 30,000 deaths each year and this figure is increasing [2]. The Hospice Palliative Care, which originated in England, aims to provide symptom alleviation and holistic care through physical, emotional, and spiritual approaches to terminally ill patients towards the end of their lives. Until 2013, the hospice care programmes available in Taiwan included 44 hospital-based inpatient hospice care wards (hospice wards), 64 hospice home-care units (regular home visits by nurses and other interdisciplinary staff), and 58 hospitals with consultation or a combined care team [3].

Terminally ill patients with cancer suffer from several physical and psychological symptoms and multiple organ failures; therefore, one of their main needs is to be comfortable and symptom-free at the end stage of their lives [4]. Consequently, the initial and essential component of palliative cancer care is to provide advanced-cancer patients with symptom relief as soon as possible in lieu of disease treatment [5, 6]. The previous studies may tell us the symptom patterns [4, 7–10] and the good effect of the intervention (ex.: the case management model, the hospital- or day care- based palliative care team…etc.) in improving symptom control and even quality of life [11–14] among advanced cancer patients. However, limited information is available regarding longitudinal assessments of symptom improvement after admission to a palliative care unit. Therefore, a clearer understanding of symptom improvement and factors associated with symptom improvement is required to provide optimal symptom relief for patients with advanced cancer. Thus, the Symptom Reporting Form was used in the present study to assess the symptom severity of patients with advanced cancer in a palliative care unit.

## Methods

### Study population

In this prospective study, participants were selected from amongst consecutive patients with advanced cancer who consented and were admitted between October 2004 and December 2009 to the palliative care unit of Kaohsiung Medical University Hospital, a medical centre in Southern Taiwan. The inclusion criteria included age older than 20 years, more than 1-week admission to the palliative care unit, and adequate level of consciousness to report symptoms in Mandarin or Taiwanese on admission and in the consecutive symptom assessments. The exclusion criteria included age younger than 20 years, passing away within one week after admission to the palliative care unit, and inadequate level of consciousness to report symptoms. The participants were receiving care from a multidisciplinary team comprising physicians, nurses, psychologists, social workers, clinical Buddhist chaplains, and volunteers. The study protocol was approved by the Kaohsiung Medical University Hospital Institutional Review Board (KMUH-IRB-20140333). Since all identifying personal information was not recorded, the review board waived the requirement for written informed consent from the patients involved.

### Assessment instrument

The assessment tool was a symptom-reporting form designed by experienced specialists, which assessed the physical and psychosocial distress by using corresponding scales (see Additional file 1). The physical and psychosocial symptoms included pain, constipation, vomiting, dyspnoea, loss of appetite, sleep disturbance, oedema, uncomfortable, depression, and anxiety, graded on a scale of 0 to 4 (0 = *none*, 1 = *mild*, 2 = *moderate*, 3 = *severe*, and 4 = *extreme*). The internal consistency of questionnaire, Coefficients of Cronbach’s α value for the baseline measurement, was 0.715. Besides, 3 experts in the hospice filed were invited to judge and modify the content of the symptom reporting form and 7 experts to assess the content validity. The average content validity index (CVI) [15] was 0.91.

### Symptom assessment and data collection

According to patient reports, the symptoms and their severities were recorded by the physicians on Days 1, 3, 5, and 7 in the palliative care unit and reviewed by the same staff members. Information regarding psychological distress, including depression and anxiety, were collected by medical staff and clinical psychologists. A consensus regarding the psychological distress rating was obtained after thorough discussion in team meetings which were held once per week. The data for this study comprised routine records, including patients’ demographic data (age, sex, primary site of cancer, and length of time in care) and the symptom reporting forms collected on Days 1, 3, 5, and 7 in the palliative care unit.

### Training of the palliative care unit staff

According to the health insurance guidelines for palliative care, all the palliative team members, including physicians, nurses, psychologists, social workers, and clinical Buddhist chaplains, were required to finish 80 h of palliative care training which included basic, advanced, and internship courses. In addition, all members were required to undergo 20 h of palliative training every year to ensure the quality of care provided.

### Statistical analysis

Descriptive statistical data were summarised as frequencies and percentages for categorical variables, medians and ranges for age and duration of palliative care received, and means (standard deviations, SD) and median (range) for symptom scores and other continuous variables. Patient age was categorised into 2 groups for easier comparisons (< 65 y and ≥ 65 y). To investigate the factors associated with the symptom reporting form scores, a generalised estimating equation (GEE) model was applied to accommodate the correlated data of repeated measurements (on Days 1, 3, 5, and 7) in the same patient. The symptom reporting score served as a continuous dependent variable, whereas age, sex, and type of cancer served as covariates. In addition, multivariate GEE models with identity links were used to calculate the adjusted mean differences and standard errors (SE) of the symptom scores and compare them with those of an appropriate reference group. A 2-tailed *P* value of < .05 indicated statistical significance. All statistical analyses were performed using the SAS software (Version 9.2; SAS Institute Inc., Cary, NC, USA).

## Results

The study participants were 824 hospitalised patients with advanced cancer; 480 (58.3 %) of whom were men. The median age was 61 years (range: 21–97 y), and the median duration in care was 20 days (7–118 d). Regarding the primary cancer type, gastrointestinal cancer was the most common cancer type (32.4 %), followed by head, neck, and oral cancer (22.4 %) and lung cancer (18.2 %) (Table 1).

**Table 1:** The baseline demographic characteristics of all participants

	Total (*N* = 824)	
	*N*	%
Age, year		
Median (Range)	61 (21–97)	
< 65	461	56.0.
≥ 65	344	41.7
missing	19	2.3
Gender		
Female	344	41.7
Male	480	58.3
Primary diagnosis		
Lung cancer	150	18.2
Gastrointestinal cancer^a^	267	32.4
Breast cancer	55	6.7
Genitourinary cancer^b^	108	13.1
Head, neck, and oral cancer	185	22.4
Other cancers^c^	59	7.2
Length of time in care		
Median (Range)	20 (7–118)	
≤ 14 days	231	28.0
15–30 days	361	43.8
31–60 days	180	21.8
> 60 days	36	4.4
Missing	16	

^a^Cancers of the gastrointestinal tract from esophagus to the rectum, pancreas, liver and gallbladder were combined to form this category

^b^Cancers of the male and female genitals and the urinary tract were combined to form this category

^c^Cancers that were not included above

Pain (78.4 %) was the most common symptom, followed by anorexia (64.4 %) and constipation (63.5 %). Regarding symptom severity, pain (mean ± SD: 1.63 ± 1.23) was the most severe symptom, followed by anorexia (1.24 ± 1.24), constipation (0.92 ± 1.05), dyspnoea (0.82 ± 1.07), and oedema (0.74 ± 1.10) (Table 2).

**Table 2:** Change in severity of symptoms among 1^st^,3^rd^,5^th^,and 7^th^ day after admission (*N* = 824)

Variable of symptom	With symptom^a^		1^st^		3^rd^		5^th^		7^th^	
Range of severity(0–4)	*N*	%	Mean (SD)	Median (range)	Mean (SD)	Median (range)	Mean (SD)	Median (range)	Mean (SD)	Median (range)
Pain	646	78.4	1.63 (1.23)	2 (0–4)	1.05 (0.99)	1 (0–4)	0.84 (0.90)	1 (0–4)	0.61 (0.81)	0 (0–4)
Constipation	463	63.5	0.92 (1.05)	1 (0–4)	0.81 (0.96)	1 (0–4)	0.75 (0.93)	1 (0–4)	0.72 (0.95)	0 (0–4)
Vomiting	313	38.0	0.62 (0.98)	0 (0–4)	0.43 (0.77)	0 (0–4)	0.34 (0.71)	0 (0–4)	0.26 (0.64)	0 (0–4)
Dyspnea	394	47.8	0.82 (1.07)	0 (0–4)	0.64 (0.92)	0 (0–4)	0.62 (0.90)	0 (0–4)	0.78 (1.12)	0 (0–4)
Anorexia	531	64.4	1.24 (1.24)	1 (0–4)	1.04 (1.14)	1 (0–4)	1.00 (1.14)	1 (0–4)	1.03 (1.28)	1 (0–4)
Sleep disturbance	356	43.2	0.67 (0.95)	0 (0–4)	0.50 (0.81)	0 (0–4)	0.46 (0.78)	0 (0–4)	0.35 (0.71)	0 (0–4)
Edema	340	41.3	0.74 (1.10)	0 (0–4)	0.67 (1.01)	0 (0–4)	0.66 (1.02)	0 (0–4)	0.71 (1.08)	0 (0–4)
Uncomfortable	364	44.2	0.67 (1.15)	0 (0–4)	0.60 (1.02)	0 (0–4)	0.57 (0.98)	0 (0–4)	0.55 (1.01)	0 (0–4)
Depression	333	40.4	0.58 (0.87)	0 (0–4)	0.47 (0.77)	0 (0–4)	0.43 (0.71)	0 (0–4)	0.31 (0.60)	0 (0–4)
Anxiety	280	34.0	0.45 (0.76)	0 (0–4)	0.38 (0.68)	0 (0–4)	0.36 (0.62)	0 (0–4)	0.25 (0.53)	0 (0–4)

^a^The score at the 1^st^ day of admission in this scale was equal to or more than 1

As mentioned previously, to investigate factors associated with the symptom reporting scores, a GEE model was applied to accommodate the correlated data of repeated measurements (on Days 1, 3, 5, and 7) in the same patient. After controlling for other factors in the multivariate GEE model, the day of palliative care administration proved a significant factor associated with all scales (all *P* ≤ 0.05), except Day 7 on the dyspnoea (*P* = .658) and oedema scales (*P* = .135) and Day 5 on the anxiety scale (*P* = .452) (Tables 3 and 4). In addition, after controlling for other factors in the multivariate GEE model, patients aged ≥ 65 years exhibited significantly lower scores on the pain, sleep disturbance, depression, and anxiety scales than did those aged < 65 years (all *P* ≤ .001). Moreover, after controlling for other factors in the multivariate GEE model, the female patients exhibited significantly higher scores on the vomiting (estimate [SD]: 0.32 [0.16] points), anorexia (0.23 [0.06] points), oedema (0.15 [0.16] points), depression (0.35 [0.11] points), and anxiety (0.77 [0.28] points) scales than did the male patients (all *P* ≤ .05) (Tables 3 and 4). Patients with gastrointestinal tract cancer exhibited higher scores on the constipation (0.21 [0.11] points), vomiting (0.90 [0.16] points), anorexia (0.59 [0.09] points), oedema (0.59 [0.16] points), depression (0.39 [0.15] points), and anxiety (0.35 [0.14] points) scales and lower scores on the dyspnoea (−0.36 [0.12] points) scale than did those with lung cancer. Patients with breast cancer exhibited higher scores on the oedema (1.43 [0.64] points) scale and lower scores on the anxiety (−0.61 [0.20] points) scale than did those with lung cancer. Furthermore, patients with genitourinary cancer exhibited higher scores on the vomiting (0.79 [0.19] points) and oedema (0.82 [0.21] points) scales and lower scores on the dyspnoea (−0.45 [0.15] points) scale than did those with lung cancer. Patients with head, neck, and oral cancer exhibited lower scores on the oedema (−1.02 [0.23] points) scale alone than did those with lung cancer. In addition, patients with other cancers exhibited higher scores on the anorexia (0.48 [0.13] points), sleep disturbance (0.58 [0.21] points), oedema (3.89 [0.54] points), uncomfortable (139.87 [0.19] points), and anxiety (3.08 [0.20] points) scales than did those with lung cancer (Tables 3 and 4).

**Table 3:** Estimates from GEE^b^ modles of indicators of pain, constipation, vomiting, dyspnea, and anorexia

	Pain			Constipation		Vomiting		Dyspnea		Anorexia	
	*N*	β(SE)^a^	*P*-value	β(SE)^a^	*P*-value	β(SE)^a^	*P*-value	β(SE)^a^	*P*-value	β(SE)^a^	*P*-value
Age, year											
< 65		Reference	-	Reference	-	Reference	-	Reference	-	Reference	-
≥ 65		−0.23 (0.06)	< 0.001	−0.19 (0.08)	0.087	−0.06 (0.12)	0.614	0.07 (0.09)	0.428	0.04 (0.06)	0.475
Gender											
Male	480	Reference	-	Reference	-	Reference	-	Reference	-	Reference	-
Female	344	0.01 (0.06)	0.828	0.18 (0.12)	0.129	0.32 (0.16)	0.046	−0.06 (0.09)	0.548	0.23 (0.06)	< 0.001
Cancer											
Lung cancer	150	Reference	-	Reference	-	Reference	-	Reference	-	Reference	-
Gastrointestinal cancer	267	0.12 (0.08)	0.146	0.21(0.11)	0.044	0.90 (0.16)	< 0.001	−0.36 (0.12)	0.002	0.59 (0.09)	< 0.001
Breast cancer	55	0.02 (0.13)	0.875	−0.20 (0.19)	0.287	0.41 (0.23)	0.075	0.12 (0.18)	0.507	−0.07 (0.15)	0.638
Genitourinary cancer	108	0.03 (0.10)	0.777	0.10 (0.14)	0.469	0.79 (0.19)	< 0.001	−0.45 (0.15)	0.002	0.19 (0.12)	0.102
Head, neck, and oral cancer	185	0.15 (0.09)	0.108	0.10(0.13)	0.452	−0.25 (0.22)	0.245	−0.22 (0.13)	0.089	−0.15 (0.13)	0.242
Other cancers	59	−0.02 (0.12)	0.836	0.09 (0.21)	0.684	−0.10 (0.34)	0.773	−0.22(0.15)	0.140	0.48 (0.13)	< 0.001
Day of palliative care											
1^st^ day	822	Reference	-	Reference	-	Reference	-	Reference	-	Reference	-
3^rd^ day	824	−0.45 (0.02)	< 0.001	−0.20 (0.08)	0.013	−0.35 (0.05)	< 0.001	−0.22 (0.03)	< 0.001	−0.18 (0.02)	< 0.001
5^th^ day	823	−0.67 (0.03)	< 0.001	−0.22 (0.04)	< 0.001	−0.47 (0.08)	< 0.001	−0.25 (0.04)	< 0.001	−0.23 (0.03)	< 0.001
7^th^ day	822	−0.98 (0.04)	< 0.001	−0.21 (0.06)	< 0.001	−1.04 (0.09)	< 0.001	−0.02 (0.05)	0.658	−0.18 (0.04)	< 0.001

^a^ Adjusted mean difference compared to referent group. ^b^GEE:generalized estimating equation

**Table 4:** Estimates from GEE^b^ modles of indicators of sleep disturbance, edema, uncomfortable, depression and anxiety

	Sleep disturbance			Edema		Uncomfortable		Depression		Anxiety	
	*N*	β(SE)^a^	*P*-value	β(SE)^a^	*P*-value	β(SE)^a^	*P*-value	β(SE)^a^	*P*-value	β(SE)^a^	*P*-value
Age, year											
< 65		Reference	-	Reference	-	Reference	-	Reference	-	Reference	-
≥ 65		−0.39 (0.11)	< 0.001	−0.26 (0.15)	0.093	−0.08(0.11)	0.510	−0.52 (0.10)	< 0.001	−0.80 (0.12)	< 0.001
Gender											
Male	480	Reference	-	Reference	-	Reference	-	Reference	-	Reference	-
Female	344	(0.07)	0.664	0.15 (0.16)	0.046	−0.06 (0.09)	0.548	0.35 (0.11)	< 0.001	0.77 (0.28)	0.005
Cancer											
Lung cancer	150	Reference	-	Reference	-	Reference	-	Reference	-	Reference	-
Gastrointestinal cancer	267	0.37 (0.14)	0.008	0.59 (0.16)	< 0.001	0.24 (0.16)	0.126	0.39 (0.15)	0.010	0.35(0.14)	0.012
Breast cancer	55	0.36 (0.37)	0.337	1.43 (0.64)	0.026	0.19 (0.28)	0.502	−0.13 (0.20)	0.527	−0.61 (0.20)	0.002
Genitourinary cancer	108	0.12 (0.19)	0.518	0.82 (0.21)	< 0.001	0.16 (0.20)	0.419	0.22 (0.19)	0.252	0.04(0.17)	0.836
Head, neck, and oral cancer	185	−0.03 (0.20)	0.881	−1.02 (0.23)	< 0.001	0.07 (0.19)	0.695	0.20(0.16)	0.215	0.34 (0.21)	0.105
Other cancers	59	0.58 (0.21)	0.006	3.89 (0.54)	< 0.001	139.87(0.19)	<0.001	0.26 (0.19)	0.166	3.08 (0.20)	<0.001
Day of palliative care											
1	822	Reference	-	Reference	-	Reference	-	Reference	-	Reference	-
3	824	−0.30 (0.04)	< 0.001	−0.11 (0.02)	< 0.001	−0.12 (0.04)	0.003	−0.20 (0.03)	< 0.001	−0.10 (0.02)	< 0.001
5	823	−0.41(0.05)	< 0.001	−0.10 (0.02)	< 0.001	−0.17 (0.05)	< 0.001	−0.28 (0.04)	< 0.001	−0.02 (0.03)	0.452
7	822	−0.66 (0.07)	< 0.001	0.10 (0.07)	0.135	−0.20 (0.06)	0.001	−0.63 (0.06)	< 0.001	−1.24 (0.05)	< 0.001

^a^ Adjusted mean difference compared to referent group. ^b^ *GEE* generalized estimating equation

## Discussion

This study demonstrated that the most severe symptoms of patients hospitalised for palliative care are pain, anorexia and constipation. In addition, the integration of hospital palliative care demonstrated favourable improvement in symptom severity. Moreover, differences in sex and primary cancer sites may contribute to varying degrees of improvement in symptom severity.

Terminally ill patients with cancer suffer from several physical and psychological symptoms and multiple organ failures; therefore, one of their main needs is to be comfortable and symptom-free at the end stage of their lives [16]. Thus, palliative cancer care essentially aims to provide such patients with symptom relief and higher quality of life [5, 17]. The findings of the present study confirmed the efficacy of palliative care based on the symptom improvement observed during the first week of the palliative unit inpatient care, except on Day 7 of dyspnoea and oedema assessment and Day 5 of anxiety assessment. Most previous studies have focused on the symptoms or symptom cluster screening in patients with advanced cancer receiving palliative care [7, 9, 18, 19]. Certain studies have conducted a longitudinal follow-up to observe the quality of life, symptom severity, and mood amongst outpatients with advanced cancer [14, 18]. The present study recruited 824 participants who were all admitted to the palliative care unit of a medical centre.

In addition, previous studies have focused on the roles of age and sex differences in symptoms improvement amongst patients with advanced cancer [9, 20–23]. Consistent with the findings of a previous systematic review of symptom prevalence [9], we observed a significant association between pain improvement and older age. Moreover, similar associations were observed with sleep disturbance, depression, and anxiety. These findings suggest that younger patients require particular attention and active monitoring during symptom management. In addition considerable sex differences were observed regarding certain symptoms: female patients reported less improvement of vomiting, anorexia, sleep disturbance, depression, and anxiety after adjustment for age, primary cancer site, and day of palliative care administration. These results are consistent with those of a large-scale study of patients who did not receive anticancer treatments, which revealed marked sex differences in the prevalence of nausea [9, 23] but is inconsistent with the results of another similar study [18]. These differences may be attributable to the different measurement methods (symptom severity vs frequency), the different treatment settings (in outpatients vs in a palliative unit care), and varying analysis methods (adjustment of other factors or repeated measurements). Therefore, additional pooled data is required from such settings to determine the effects of age and sex on symptom improvement in such a patient population.

Moreover, our results helped identify the crucial aspects of symptom improvement for different primary cancer sites. Regarding pain, no significant differences were observed between the different types of cancer during the first week of admission. Gastrointestinal symptoms, including constipation, vomiting, and anorexia, were the most common and severe problems in patients with advanced cancer, particularly those with advanced gastrointestinal cancer; these symptoms may be attributable to refractory ascites or intra-abdominal masses or may be the side effects of opioid drugs administered to such patients. Dyspnoea is a concerning symptom and is more challenging to relieve in patients with advanced lung cancer. It may result from the destruction of lung tissue because of the growth and spreading of cancer cells. In addition, improvement of sleep disturbance was significantly lower amongst patients with advanced gastrointestinal cancer than amongst those with advanced lung cancer. Compared with patients with advanced lung cancer, management of lymphoedema was more challenging amongst those with advanced gastrointestinal, breast, and genitourinary cancer but easier amongst those with head, neck, and oral cancer. However, the varying sites of lymphoedema amongst patients with different types of cancer required further investigation to facilitate efficient management. Improvement of mood states, including depression and anxiety, was more challenging amongst patients with advanced gastrointestinal cancer than amongst those with advanced lung cancer ones. Patients with advanced breast cancer exhibited greater control of anxiety than did those with lung cancer; the reasons for this difference may be complicated because of other underlying physical symptoms (eg, dyspnoea).

The conditions of patients with advanced cancer may rapidly deteriorate during their terminal phase, and symptoms often worsen rapidly, suddenly, and unexpectedly. Thus, rapid and effective management of these symptoms is essential to ensure adequate improvement and a high ‘quality of death’. Therefore, the symptom severity assessment of patients with advanced cancer in palliative care units may provide additional valuable information regarding this crucial aspect of quality of care.

This was an observational study which evaluated clinical experiences and thus had considerable limitations. First, the symptom assessment checklist was a relatively short instrument which assessed only 10 symptoms. Second, assessment of the symptom scale scores every 2 days may be too close. However, this study aimed to explore the levels of symptom improvement after admission to the palliative care unit, where the palliative care team could resolve the symptoms as soon as possible. In addition, because the cancer progression in such patients may alter unexpectedly, we used a simple instrument to closely assess the improvement in symptom severity. Third, there was no information of the individual co-morbidity, which may be associated with distressing symptoms among advanced cancer patients. Fourth, this study recruited only advanced cancer patients with adequate level of consciousness to report symptoms and was conducted in one palliative care unit of a medical center, which may limit the generalization for all terminal patients and all palliative care units. A consistent symptom assessment tool for the routine quality indicators of the palliative care units is needed in the future.

## Conclusion

This study confirmed considerable symptom improvement during the first week of palliative unit inpatient care, except on Day 7 of dyspnoea and oedema assessment and Day 5 of anxiety assessment. Improvement of pain, sleep disturbance, depression, and anxiety were significantly associated with older age. In addition, female patients with advanced cancer reported lower symptom improvement of vomiting, anorexia, sleep disturbance, depression, and anxiety than did the corresponding male patients. In addition, patients with different types of advanced cancer exhibited varying degrees of symptom improvement.

## Additional file

Additional file 1: The Symptom Reporting Form. (DOC 30 kb)

## Abbreviations

GEE model: a generalised estimating equation model
Estimate [SD]: estimate [standard deviation]
