# Supplementary material for: Symptom severity of patients with advanced cancer in palliative care unit: longitudinal assessments of symptoms improvement
Source: BMC Palliat Care. 2016 Mar 11;15:32. doi: 10.1186/s12904-016-0105-8 (PMC4787050; doi:10.1186/s12904-016-0105-8)
Supplement: Additional file 1: — The Symptom Reporting Form. (DOC 30 kb) [file 12904_2016_105_MOESM1_ESM.doc]

Additional file 1: The Symptom Reporting Form

Ward no.:

Chart no.: Name: Sex: Age:

Birthday:

Primary cancer:

Admission date:

Assessment date: 1st___ 3rd___ 5th___ 7th___ of admission date

Assess the patient about:

Q1: “Over the past 2 days, has the patient been affected by pain?”

Area?

[ ] 0 No, not at all

[ ] 1 Mild, but not bothered to be rid of it

[ ] 2 Moderate, limits some activity

[ ] 3 Severe, activities or concentration markedly affected

[ ] 4 Extreme, unable to think of anything else

[ ] head & neck , [ ] chest , [ ] abdomen , [ ] pelvic , [ ] legs, [ ] others_______

Q2: “Over the past 2 days, has the patient been affected by constipation?”

[ ] 0 No, not at all

[ ] 1 Mild

[ ] 2 Moderate

[ ] 3 Severe

[ ] 4 Extreme

Q3: “Over the past 2 days, has the patient been affected by nausea/vomiting?”

[ ] 0 No, not at all

[ ] 1 Mild

[ ] 2 Moderate

[ ] 3 Severe

[ ] 4 Extreme

Q4: “Over the past 2 days, has the patient been affected by dyspnoea?”

[ ] 0 No, not at all

[ ] 1 Mild

[ ] 2 Moderate

[ ] 3 Severe

[ ] 4 Extreme

Q5: “Over the past 2 days, has the patient been affected by anorexia?”

[ ] 0 No, not at all

[ ] 1 Mild

[ ] 2 Moderate

[ ] 3 Severe

[ ] 4 Extreme

Q6: “Over the past 2 days, has the patient been affected by sleep disturbance?”

[ ] 0 No, not at all

[ ] 1 Mild

[ ] 2 Moderate

[ ] 3 Severe

[ ] 4 Extreme

Q7: “Over the past 2 days, has the patient been affected by edema?”

[ ] 0 No, not at all

[ ] 1 Mild

[ ] 2 Moderate

[ ] 3 Severe

[ ] 4 Extreme

Q8: “Over the past 2 days, has the patient been affected by other uncomfortable?”

[ ] 0 No, not at all

[ ] 1 Mild

[ ] 2 Moderate

[ ] 3 Severe

[ ] 4 Extreme

Q9: “Over the past 2 days, has the patient been affected by depression mood?”

[ ] 0 No, not at all

[ ] 1 Mild

[ ] 2 Moderate

[ ] 3 Severe

[ ] 4 Extreme

Q10: “Over the past 2 days, has the patient been affected by anxiety?”

[ ] 0 No, not at all

[ ] 1 Mild

[ ] 2 Moderate

[ ] 3 Severe

[ ] 4 Extreme
